# Preenrichment with Adipose Tissue-Derived Stem Cells Improves Fat Graft Retention in Patients with Contour Deformities of the Face

**DOI:** 10.1155/2019/5146594

**Published:** 2019-11-20

**Authors:** Muhammad M. Bashir, Muhammad Sohail, Fridoon J. Ahmad, Mahmood S. Choudhery

**Affiliations:** ^1^Plastic Surgery Department, King Edward Medical University/Mayo Hospital, Lahore, Pakistan; ^2^Tissue Engineering and Regenerative Medicine Laboratory, Department of Biomedical Sciences, King Edward Medical University, Lahore, Pakistan

## Abstract

Quick absorption of adipose tissue grafts makes the outcomes less satisfactory for clinical applications. In the current study, adipose tissue grafts were mixed with adipose tissue-derived stem cells (ASCs) to improve retention of adipose tissue grafts and to make the clinical outcomes of fat grafting more reliable. Adipose tissue was either injected alone (conventional group) or mixed with ASCs (stem cell group) before injection. In both groups, adipose tissue was injected at the site of contour throughout layers of tissues till visual clinical symmetry with the opposite side was achieved. The volume of injected fat graft was measured after 72 hours and 6 months using a B-mode ultrasound device connected with a 12 MH frequency probe. The percentage reduction in the volume of injected fat, physician satisfaction scores (Ph-SCs), and patient satisfaction scores (P-SCs) were also recorded. After 6 months, there was significantly lower fat absorption in the stem cell group as compared to the conventional group. Mean physician and patient satisfaction scores were significantly improved in the stem cell group. No significant adverse effects were noted in any patient. Significantly lower absorption of graft due to the use of ASCs improves the clinical outcomes of conventional fat grafting for contour deformities of the face. The current preenrichment strategy is noninvasive, safe and can be applied to other diseases that require major tissue augmentation such as breast surgery. This trial is registered with NCT02494752.

## 1. Introduction

Autologous fat grafting is a frequently employed procedure in cosmetic and reconstructive surgery. Fat is a versatile filler for treating contour irregularities of the face brought about by congenital disorders, acquired diseases, and traumatic and developmental deformities. Unlike many other fillers of synthetic origin, fat is easy to procure with minimal donor site morbidity. Additionally, it is frequently available as autologous and thus without immunogenicity issue. Moreover, its soft and dynamic nature makes it useful especially for cosmetic and reconstructive surgery [1]. Although adipose tissue grafting is a well-known technique to correct contour irregularities, quick absorption of fat at the site of application is a major concern for patients as well as clinicians [2]. The rate of fat absorption may reach up to 90% due to hypoxic and ischemic environment after transplantation. Clinically, this unreliability produces unsatisfactory and suboptimal final clinical outcomes, and therefore, multiple sessions of fat grafts are required, making this procedure expensive and lengthy [2, 3]. In order to improve survival of transplanted adipose tissue graft, alternative approaches are required. In the current study, autologous fat graft was preenriched with culture-expanded adipose tissue-derived stem cells (ASCs) to enhance retention of transplanted graft and to make clinical outcomes more reliable.

Stromal vascular fraction (SVF) of adipose tissue is a heterogeneous cell population containing blood cells, fibroblasts, pericytes, endothelial cells, and ASCs. Studies indicate that ASCs have angiogenic, antiapoptotic, immunosuppressive, and immunomodulatory properties that make them ideal candidates for clinical use [4]. Recent animal studies have demonstrated improved survival and retention of grafted fat when adipose tissue grafts were preenriched with ASCs [5]. If enrichment of fat graft with ASCs can decrease its absorption rate, this innovative strategy can make fat transfer a more reliable option for soft tissue augmentation. This can definitely improve final clinical outcomes at lesser cost and reduce donor site morbidity.

The current study is aimed at evaluating the effect of ASCs on survival and retention of adipose tissue grafts in patients of contour deformities of the face. Autologous adipose tissue was harvested, processed, and either injected alone or mixed with ex vivo expanded ASCs at the site of face contour. Reduction in the volume of injected graft, physician satisfaction scores (Ph-SCs), and patient satisfaction scores (P-SCs) were recorded. Results indicated that preenrichment with ASCs significantly reduces fat absorption at the site of application and improves the clinical outcomes of conventional fat grafting as indicated by Ph-SCs and P-SCs. To the best of our knowledge, this is the first study that was performed on actual patients of contour deformities of the face to show the effect of preenrichment of adipose tissue grafts with ASCs. This technique will open a new avenue not only for the augmentation of contour deformities of the face but also for other conditions that require large tissue augmentation procedures such as breast reconstruction.

## 2. Material and Methods

**Figure 1:**
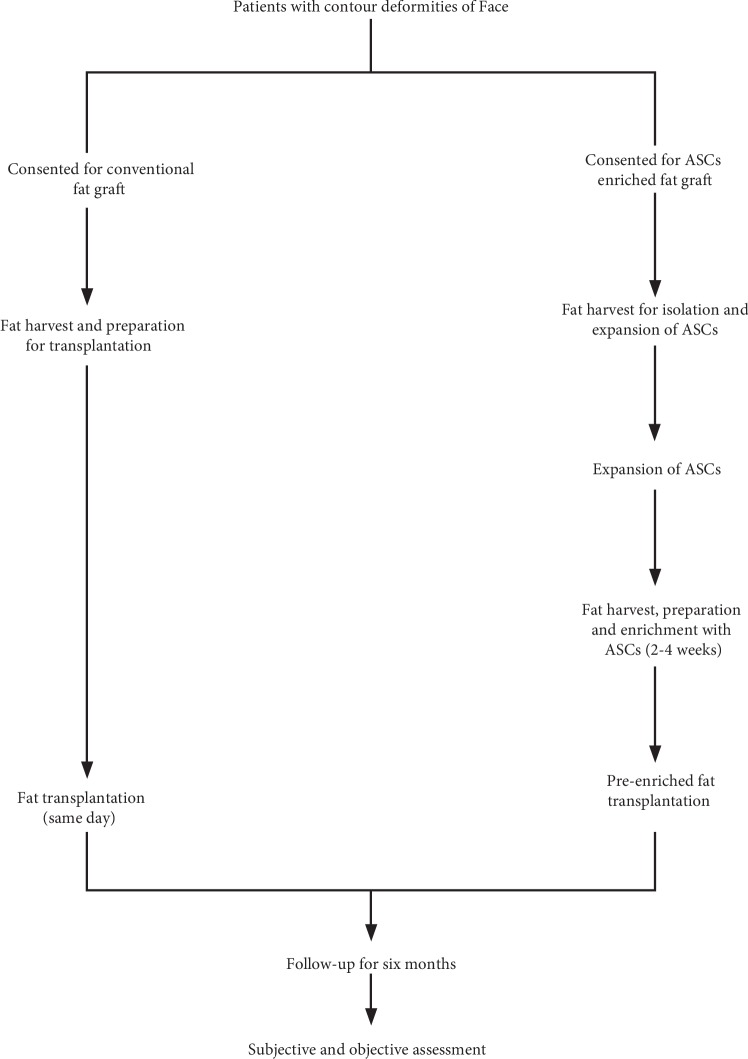
Flow chart showing the management plan of patients with contour deformities of the face.

It was a quasi-experimental study conducted at “Department of Plastic & Reconstructive Surgery” and “Tissue Engineering and Regenerative Medicine Laboratory” Department of Biomedical Sciences, King Edward Medical University/Mayo Hospital, Lahore. Thirty-seven patients with congenital or acquired contour deformities of the face were enrolled consecutively from September 2015 to September 2017. The patients with contour deformities in skin-grafted areas and where the skin was adherent to the facial skeleton were excluded [6]. Demographic and clinical data of patients was collected after obtaining informed written consent. One week before the surgery, patients in each group were advised to stop taking aspirin, alcohol, or any other herbal medications. The protocols used in the study were approved by the IRB (Institutional Review Board) of King Edward Medical University, Lahore, Pakistan (letter # 229/RC/KEMU). The study was performed according to “The Code of Ethics of the World Medical Association (Declaration of Helsinki),” and the trial was registered at ClinicalTrials.gov (NCT02494752). Both methods (fat graft only or stem cells mixed with fat graft) were offered to patients. Patients giving consent for traditional fat grafting (conventional group) underwent fat harvest, preparation, and transplantation on the same day, while patients giving consent for ASC-enriched fat grafting (stem cell group) underwent fat harvest two times, first to isolate and expand ASCs in vitro and second (after 2-3 weeks) to preenrich fat grafts with culture-expanded ASCs. The full overview of the study is given in Figure 1.

### 2.1. Fat Harvest and Processing

Depending on patient desire and accessibility, fat was harvested from either the abdomen or the lateral side of the thigh as described previously by our group [6]. Briefly, under local or general anesthesia, fat harvest area was infiltrated with tumescent solution consisting of 0.4% lidocaine and 1 : 1000.000 epinephrine. Fat was harvested using a 3 mm, two-hole, blunt cannula attached to a 10 ml Luer-Lok syringe. The plunger of a 10 cc syringe was pulled back only a few milliliters during suctioning to evade unnecessary negative pressure and to avoid fat cell rupturing. The required amount to fill the contour deformity was harvested accordingly on the basis of clinical judgment.

### 2.2. Preparation of Fat for Transplantation

**Figure 2:**
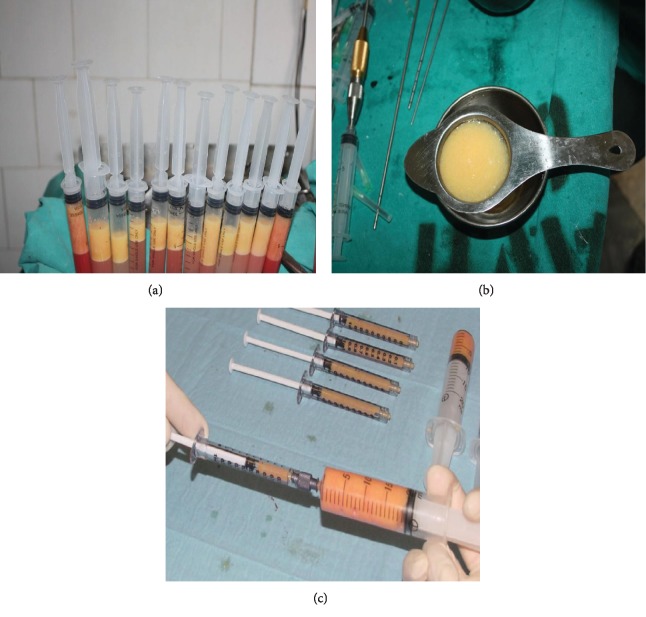
(a) Syringes standing vertically to allow gravity sedimentation. (b) The filtration of fat through the common strainer to concentrate the fat and to separate it from oil and debris. (c) The transfer of fat from 10 cc syringes to 1 cc syringes for transplantation.

The fat tissue for transplantation was prepared by using the methods of Bashir et al. [6]. Briefly, to separate the fat from liquid portion, 10 ml Luer-Lok syringes were kept vertically for 5-10 minutes (Figure 2(a)). The tumescent fluid and blood were drained out from the bottom. To further purify fat from debris and oil, the remaining fat in the syringes was passed through a common strainer (Figure 2(b)). The residue was then washed with 0.9% saline solution. After washing, purified fat was collected in 10 cc Luer-Lok syringes and was transferred to 1 cc syringes for transplantation (Figure 2(c)).

### 2.3. Isolation of Adipose Tissue-Derived Stem Cells (ASCs)

For ASC isolation, fat tissue was harvested under local anesthesia and processed as described previously by us [6, 7]. Fat tissue (20 ml-30 ml) was harvested under sterilized conditions and transferred to a certified laboratory approved by the Human Organ Transplantation Authority (HOTA) for processing and isolation of ASCs. ASCs were isolated and culture expanded by enzymatic digestion using Good Manufacturing Practice- (GMP-) grade reagents. After 2 weeks, the ASCs were isolated by enzymatic digestion as described previously by Choudhery et al. [7]. Briefly, lipoaspirate was treated for 30 minutes with 0.2% collagenase type IV (Sigma, USA) to digest the tissue slurry. The patient serum was used to neutralize collagenase activity. The cell suspension was passed through a 70 *μ*m strainer to remove undigested tissue pieces and debris. The filtered solution was centrifuged at 1000 rpm for 10 minutes at 4°C to obtain cells in a pellet. The pellet was washed with phosphate-buffered saline (PBS), and cell suspension was again centrifuged at 1000 rpm for 10 minutes. The cells were cultured in complete growth medium, i.e., MEM-alpha (Thermo Scientific, USA), supplemented with 1% each of nonessential amino acids, streptomycin/penicillin solution, and 5% autologous serum. The cells were resuspended in complete medium and were plated in 25 cm^2^ culture flasks at 37°C/5% CO_2_ with humidity. After 24 hours, the nonadherent cells were discarded and fresh medium was added. Medium was changed twice a week thereafter until the cells became confluent. The cells were trypsinized and cultured in 75 cm^2^ culture flasks. At 80%-90% confluence, cells were dissociated using trypsin-EDTA and counted using a hemocytometer. The cells at passage one were used for preenrichment of adipose tissue graft. During this period, the cells were regularly monitored under a microscope for any type of contamination or morphological changes.

### 2.4. Fat Grafting at Contour Deformities of the Face

In patients of the conventional group, purified fat collected in 10 cc syringes was transferred to 1 cc syringes (using a two-way connector) for transplantation to the recipient area. In patients of the stem cell group, on the day of fat transplantation, fat was again harvested, prepared, and purified as described above for the conventional group. The ex vivo expanded ASCs (10^6^ cells per ml of fat) were mixed with the purified fat and transferred to 1 cc syringes (Figure 2(c)). In both groups, the fat was injected through a 1.5 mm blunt-tip cannula, with a lateral opening using small stab incisions. Fat was placed gently during the withdrawal of the cannula. Fat was placed in small fractions at different depths of soft tissue starting from just above the periosteum and moving superficially [8]. End point of lipofilling was achieved by visual clinical symmetry with the opposite side. Quantity of fat transferred was noted in milliliters. Patients were given intravenous first-generation cephalosporin for 72 hours and discharged on oral antibiotic within a week. The patients were followed up at monthly intervals for six months.

### 2.5. Assessment

Both subjective and object assessments were performed to compare the outcomes of transplantation in both groups. Mean ± SD volume of injected fat per procedure was measured in milliliters (ml) as described [6]. The number of fat grafting sessions and total volume of fat injected to achieve clinical symmetry per case were also noted.

### 2.6. Surgeon Satisfaction and Patient Satisfaction Assessment

It was performed for clinical symmetry (comparing the affected side to the unaffected side) and overall appearance. It was done by clinical examination and comparing the preoperative and six-month postoperative (after the first and final fat graft sessions) photographs [6]. Two plastic surgeons (blinded to group allocation) independently rated postoperative appearance using a five-point scale as (1) very unsatisfied (gross asymmetry and severe deformity), (2) unsatisfied (significant asymmetry and deformity), (3) neither satisfied nor unsatisfied (perceptible asymmetry and deformity), (4) satisfied (hardly perceptible asymmetry and deformity), and (5) extremely satisfied (no asymmetry and normal appearance). The same surgeons rated each patient at both intervals. Similarly, using the same five-point scale, the patients were asked to comment about symmetry (comparing the affected side to the unaffected side) and overall appearance by comparing the preoperative and six-month postoperative (after the first and final fat graft sessions) photographs.

### 2.7. Objective Assessment

For objective assessment, we performed ultrasonography as described previously [6] using a B-mode ultrasound device and a 12 MH high-frequency probe (Sonoace X4, Medison). Each patient was placed in upright position, and a thick layer of water-based gel was put on the treated area for transmission of ultrasound waves. The transducer probe was placed on the assessment site perpendicular to the skin surface. The correct transducer position was determined to differentiate noise from proper ultrasound reflections. Linear measurements of soft tissue thickness of the treated area were performed. The thickness of the subcutaneous tissue was measured in millimeters in triplicate, and the mean of three readings was used for analysis. The first measurement of soft tissue thickness was done 72 hours after transplantation in both groups. In order to have a reproducible measurement in subsequent examinations, the operator noted down and marked precise anatomical landmark points with an indelible marker, saving a digital image for future reference. Ultrasonography was repeated after six months by the same operator who measured the subcutaneous thickness of the treated area. The difference in measurements taken at 72 hours postoperatively and at six months after the first fat graft session was noted down as percentage reduction in fat graft volume.

### 2.8. Regrafting

The need for regrafting the affected area was assessed based on patient and physician satisfaction. Fat grafting session was repeated following the same protocol in cases where symmetry was distorted. Patients were photographed on each follow-up under standard conditions of light, distance, views, and camera make.

### 2.9. Complications

All the patients were observed for possible complications such as infection (swelling and redness of the skin that feels hot and tender), bruising (skin discoloration due to rupture of small blood vessels), swelling (localized enlargement of the treated area due to inflammatory fluid accumulation), skin necrosis (loss of skin and subcutaneous tissue), hematoma (abnormal collection of blood), seroma (abnormal collection of fluid), and uneven skin texture (irregular, pebbly, and rough skin surface) [6].

### 2.10. Statistical Analysis

Data were analyzed using SPSS version 16. Qualitative variables like gender, etiology, fat harvesting site, and complications of procedure were expressed as proportions. Quantitative variables like age, patient and physician assessment scores, volume of fat injected in one procedure, total volume of fat injected per case, and percentage reduction in fat graft volume were expressed as mean (SD). In order to compare quantitative data, normality was tested using the one-sample Kolmogorov-Smirnov test. We applied the independent sample *t*-test (mean ± SD was compared) for variables with normal distribution and the Mann–Whitney *U* test for variables which did not have normal distribution (median ± IQR was compared). *P* value ≤ 0.05 was considered significant.

## 3. Results

The total number of patients in two groups was 37. The mean age of patients was 21 ± 5 and 30 ± 11 years with 17 (81%) and 9 (56%) females in the conventional and stem cell groups, respectively. The most common indication for fat grafting was idiopathic hemifacial atrophy in 10 (48%) and 9 (56%) patients followed by congenital craniofacial microsomia in 5 (24%) and 4 (25%), posttraumatic deformity in 5 (24%) and 2 (13%), and postinfective deformity in 1 (4%) and 1 (6%) patients in the conventional and stem cell groups, respectively. Patients underwent a total of 67 fat grafting sessions (51 sessions for 21 patients in the conventional group) and 16 sessions (one each for 16 patients in the stem cell group) over a period of 24 months.

**Figure 3:**
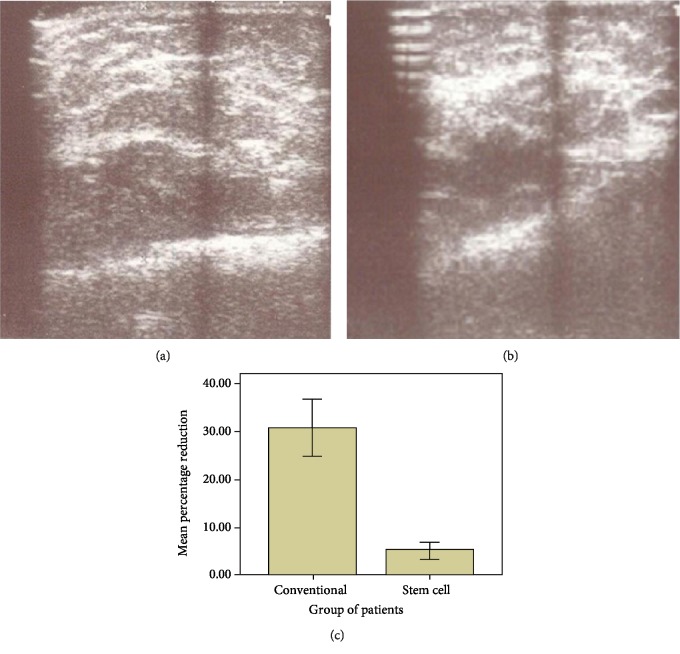
Representative ultrasonic measurement of soft tissue thickness (a) after 72 hours and (b) 6 months after transplantation. (c) Comparison of mean percentage reduction of fat absorption in both groups six months after the 1^st^ fat graft session.

There was no statistically significant difference in distribution of patients according to gender, preoperative soft tissue thickness, and mean volume of fat transferred per session (Table 1). However, there was a statistically significant difference in mean age of the patients and number of fat graft sessions in two groups (Table 1). After 6 months, there was a significantly lower fat absorption as evident by percentage reduction of 5 ± 4.4 in the stem cell group as compared to 31 ± 13 in the conventional group (*P* value < 0.001).  Figure 3(a) shows ultrasonic measurement of soft tissue thickness after 72 hours, and Figure 3(b) shows measurements 6 months after injections. Comparison of mean percentage reduction of fat absorption in both groups six months after the 1^st^ fat graft session has been shown in Figure 3(c).

**Figure 4:**
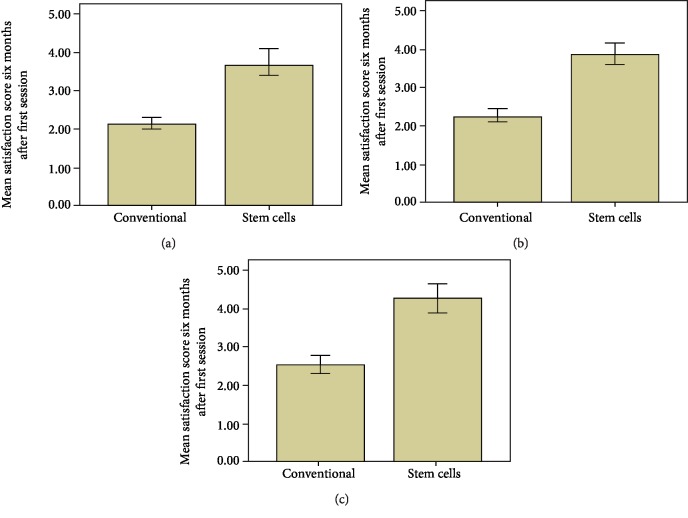
Comparison of mean (a) physician 1 and (b) physician 2 satisfaction score in both groups six months after the 1^st^ fat graft session. (c) Comparison of mean of patient satisfaction score in both groups six months after the 1^st^ fat graft session.

**Figure 5:**
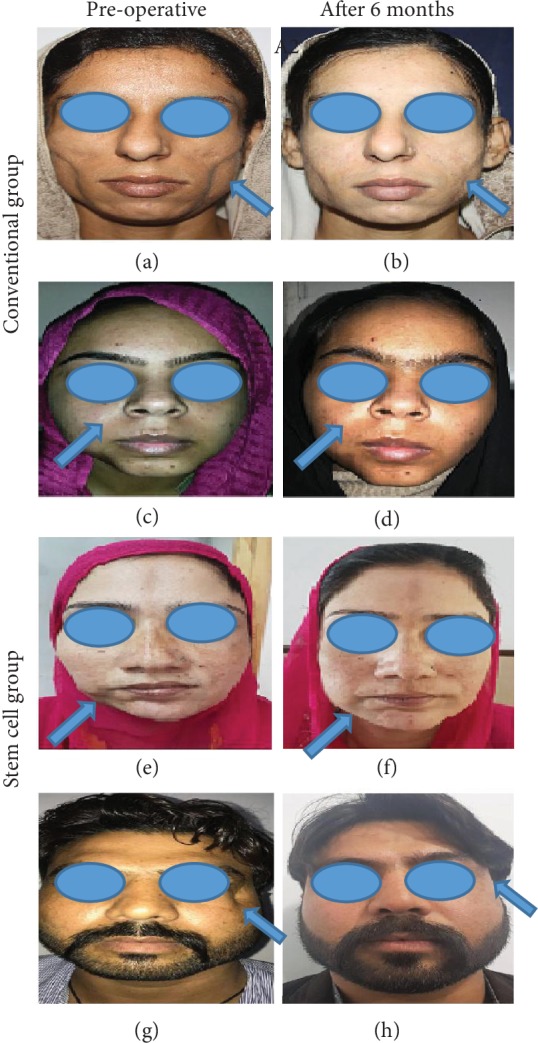
Representative picture showing preoperative and postoperative view of patients in (a–d) the conventional group and (e–h) the stem cell group. (a, c) Preoperative view of patient 1 (with facial contour deformity involving bilateral cheeks) and patient 2 (with facial contour deformity involving right mandibular area), respectively. (b, d) Fat absorption six months after the 1^st^ session of conventional lipofilling. (e, g) Preoperative view and (f, h) postoperative view of patients in the stem cell group. (e) exhibits preoperative view of a patient with contour deformity of the right mandibular area, and (f) shows postoperative view six months after a single session of ex vivo expanded ASC-enriched lipofilling. (g) represents preoperative view of a patient with contour deformity of the face near the eye, and (f) shows postoperative view six months after a single session of ex vivo expanded ASC-enriched lipofilling in some patients.

Mean patient and physician satisfaction scores have been depicted in Figure 4. Mean physician satisfaction score was 2.14 ± 0.36 in the conventional group and 3.69 ± 0.79 in the stem cell group. Similarly, mean patient satisfaction score (2.52 ± 0.521) in the conventional group was lower as compared to that in the stem cell group (4.25 ± 0.68). Overall, as shown in Figures 4(a)–4(c), there were significantly high patient and physician satisfaction scores in patients transplanted with ASC-enriched fat. Representative pictures of patients in both groups clearly show less absorption of fat when adipose tissue graft was enriched with culture-expanded ASCs (Figure 5).

None of the patients had serious complications during or after procedure; however, minor complications noted were comparable in both groups. For example, postoperative swelling was observed after in all procedures which settled on its own, and bruising was observed after 36 (71%) sessions which resolved in 2-3 weeks. Recipient site cellulitis developed in three (6%) cases which settled at two weeks with oral antibiotics in two cases, and in one case, it progressed to discharge of pus from the injection site that required IV clarithromycine for one month based on culture report. In the stem cell group also, the most common complication was postoperative swelling observed in all (100%) patients that also resolved in 2-3 weeks. Only one (6%) patient had recipient site cellulitis that settled at two weeks with oral antibiotics. Bruising was observed in 11 (69%) patients. None of the patients had seroma, hematoma, or skin necrosis in both groups.

## 4. Discussion

In the present study, clinical outcomes of treatment of contour deformities of the face with conventional fat grafting were compared with ex vivo expanded ASC-enriched fat grafting prospectively. To the best of our knowledge, it is the first study that was performed on patients of facial contour deformities using enriched ASCs. The results indicate that preenrichment of adipose tissue graft with ASCs significantly reduces fat resorption and thus makes better the clinical outcomes in patients of contour deformities of the face. Although autologous fat grafting is a safe and effective technique for restoring volume in contour deformities of the face, significant resorption is observed in several studies including the current study (Table 1). This finding is noteworthy owing to the fact that mean preoperative soft tissue thickness, volume of transferred fat, and soft tissue thickness 72 hours after the first fat graft session were similar in both groups (Table 1). Patient and physician satisfaction has a pivotal role especially in facial plastic surgery. In our study, patient and surgeon satisfaction 6 months after the first fat graft session was significantly higher in the stem cell group as compared to the conventional group (Figure 4). Majority of the patients in the conventional group underwent more than one fat graft session due to high volume of fat absorbed over time; however, none of the patients in the stem cell group required a second session (Table 1).

Autologous fat grafting reported by Neuber in 1893 has now become the most widely accepted plastic surgery procedure for soft tissue augmentation, reconstruction, and rejuvenation [9, 10]. A large number of technical details of autologous fat grafting had been described claiming superiority of one over the other [4, 11]. Majority of studies found no difference in the number of adipocytes in fat harvested from the abdomen, thigh, flank, or around knee [12]. Different studies comparing harvesting methods have found a greater number of viable adipocytes with better cellular function in syringe-aspirated fat graft. Similarly, many studies comparing processing of fat graft by gravity and filtration followed by washing versus centrifugation showed no effect on cellular viability [13]. In the current study, adipose tissue was harvested with less traumatic syringe aspiration method to gain the maximum number of viable cells. Harvested adipose tissue was minimally handled and quickly processed with gravity and filtration (Figure 2). Small aliquots were injected throughout layers of tissue to improve long-term cell survival. Additionally, mean and total volume of injected fat in a single session were comparable to those delineated in the literature [14]. In recent reviews, outcomes of fat grafting determined by a multifactorial process showed highly variable retention rates (20% to 95%) [2, 3, 14]. We also found highly variable reduction in soft tissue thickness (12%-66%) in the conventional group at 6 months. Interestingly, however, in the stem cell group, reduction in soft tissue thickness was far less (1.06% to 12.62%) than that in the conventional group (Figure 3). This difference in outcome can be attributed to the addition of ex vivo expanded ASCs to the fat grafts in the stem cell group (Figures 3(c) and 5). In another study, fat samples enriched with SVF exhibited better survival and retention [15]. The considerable difference in resorption rates highlights the fact that SVF contain heterogenous cell population including ASCs [16]. Furthermore, the exact number of ASCs present in SVF varies between both individual patients and the body sites from which lipoaspirate is obtained [17]. The rationale for use of ASCs is to obtain a potentially larger number of more regenerative cells (ASCs) [18]. The ASC-enriched fat grafts displayed higher amounts of transplanted adipose tissue and newly formed connective tissue and less necrotic tissue after 121 days [19]. Although previous studies have used different numbers of cells to preenrich adipose tissue graft [18, 19], in the current study, favorable results were obtained by adding 10^6^ ASCs per ml of fat. This number of cells can easily be obtained in the initial passages of cells.

ASCs have the capacity to differentiate into multiple cell lines including chondrocytes, adipocytes, myocytes, and osteoblasts [20, 21]. ASCs also produce different secretomes (“vascular endothelial growth factor,” “basic fibroblast growth factor,” and “hepatocyte growth factor”) having provasculogenic and immune modulatory properties [20]. Various in vivo models have analyzed the beneficial effects of ASCs in diabetic wounds, ischemic flaps, and replicas of cardiac ischemia [22, 23]. Recent studies have identified ASC-induced neovasculogenesis as a main contributor to survival of fat grafts [14, 24]. We propose that transferred fat may act as a natural scaffold and temporary filler to restore the volume immediately while ASCs will start participating in multiple parameters of tissue regeneration. This model supports the “host replacement theory” that has been put forward to describe how fat grafts survive after they are transplanted [18]. In this study, both photometric evaluation and volumetric assessment (soft tissue thickness of treated areas) were performed using B-mode ultrasonography [6], which is noninvasive, cost-effective, and easily available at the institution and avoids radiation exposure [10]. In the current study, frequencies of postoperative complications were comparable to those described in the literature. Swelling was observed in all patients which settled in 3-4 weeks. Infection was managed conservatively. The present study has few limitations. This is a single-center study limiting its generalizability. Furthermore, patients were not selected randomly with possibility of selection bias. Another limitation was small sample size of our study and short follow-up of six months. Although it was not possible to determine the long-term fate of transferred fat, previous studies have shown progressive reduction of the soft tissue thickness within the first 3 months after initial operations with stabilization of these rates from 3 to 6 months postoperatively. The suggested superiority of ex vivo expanded ASC-enriched lipotransfer over traditional lipofilling should be investigated further.

## Figures and Tables

**Table 1:** 

	Conventional group (*n* = 21)	Stem cell group (*n* = 16)	*P* value
*Patient characteristics*			
Males	4	7	0.151^a^
Females	17	9	
Age (years) (mean ± SD)	21 ± 5	30 ± 11	0.004^b^

*Number of sessions and volume injected*			
Number of fat graft sessions (mean ± SD)	2 ± 1	1 ± 0	<0.001^c^
Volume of fat transferred per session (ml) (mean ± SD)	26 ± 14	29 ± 14	0.524^b^

*Assessment*			
Soft tissue thickness (mm)			
(i) Preoperative (mean ± SD)	5 ± 2	5 ± 4	0.602^b^
(ii) Postoperative 72 hours after the first fat graft session (mean ± SD)	19 ± 8	24 ± 8	0.072^b^
(iii) Postoperative 6 months after the first fat graft session (mean ± SD)	13 ± 6	23 ± 9	<0.001^b^

^a^Chi-square test, ^b^independent sample *t*-test, and ^c^Mann–Whitney *U* test.

## Data Availability

The data used to support the findings of this study are included within the article.

## Abbreviations

ASCs: Adipose tissue-derived stem cells
SVF: Stromal vascular fraction
IRB: Institutional Review Board
GMP: Good Manufacturing Practice
HOTA: Human Organ Transplantation Authority
PBS: Phosphate-buffered saline
MEM: Minimum essential medium
Ph-SCs: Physician satisfaction scores
P-SCs: Patient satisfaction scores.
